# Integration of single sample and population analysis for understanding immune evasion mechanisms of lung cancer

**DOI:** 10.1038/s41540-023-00267-8

**Published:** 2023-02-10

**Authors:** Xiong Li, Xu Meng, Haowen Chen, Xiangzheng Fu, Peng Wang, Xia Chen, Changlong Gu, Juan Zhou

**Affiliations:** 1 School of Software, East China Jiaotong University, Nanchang, 330013 China; 2 College of Computer Science and Electronic Engineering, Hunan University, Changsha, China

**Keywords:** Computational biology and bioinformatics, Systems biology

## Abstract

A deep understanding of the complex interaction mechanism between the various cellular components in tumor microenvironment (TME) of lung adenocarcinoma (LUAD) is a prerequisite for understanding its drug resistance, recurrence, and metastasis. In this study, we proposed two complementary computational frameworks for integrating multi-source and multi-omics data, namely ImmuCycReg framework (single sample level) and L0Reg framework (population or subtype level), to carry out difference analysis between the normal population and different LUAD subtypes. Then, we aimed to identify the possible immune escape pathways adopted by patients with different LUAD subtypes, resulting in immune deficiency which may occur at different stages of the immune cycle. More importantly, combining the research results of the single sample level and population level can improve the credibility of the regulatory network analysis results. In addition, we also established a prognostic scoring model based on the risk factors identified by Lasso-Cox method to predict survival of LUAD patients. The experimental results showed that our frameworks could reliably identify transcription factor (TF) regulating immune-related genes and could analyze the dominant immune escape pathways adopted by each LUAD subtype or even a single sample. Note that the proposed computational framework may be also applicable to the immune escape mechanism analysis of pan-cancer.

## Introduction

In 2020, there were 9.96 million cancer deaths worldwide, including 1.8 million deaths from lung cancer, far exceeding other cancer types^1,2^. Lung adenocarcinoma (LUAD) is the main pathological type, accounting for >40% of lung cancers. At present, the occurrence and evolutionary mechanism of LUAD are still unclear^3^, and the tumor heterogeneity makes it difficult to apply personalized medicine, or even to accurately assess the prognosis of each patient^4^.

The tumor microenvironment (TME) is the “soil” of lung cancer cells and plays a vital role in the survival and evolution of tumor cells. Emerging evidence shows that the components in TME can define the immunophenotype of cancer and can be used for prognostic evaluation of patients. The TME is composed of various immune cells, stromal cells (including mesenchymal cells and endothelial cells), extracellular matrix molecules, and various cytokines^5,6^. In TME, cytotoxic immune cells with low infiltration level or low activity level are difficult to carry out effective immune attacks on tumor cells, leading to uncontrolled growth, evolution, and even metastasis of tumor cells. Therefore, it is necessary to deeply understand the components of TME of different LUAD subtypes and the interactions between immune cells and tumor cells in the TME.

The gene expression level is regulated by a combination of transcription factors (TFs). The studies on transcription regulation have been conducted for >30 years, and lots of landmark models have been designed^7–9^. The regulation of TFs may be the basis for the differential expression of target genes. Therefore, based on the expression profile of genes and TFs, we can use machine learning methods to build gene regulatory network (GRN). For example, Emad, A. and Sinha, S. proposed a transcriptional regulatory network which revealed the relationship between the expression of disease-related genes and cell health^10^. However, we still lack of comprehensive understanding how TFs regulate immune-related genes (IR genes)^11^, let alone on a single sample level. Constructing a regulatory network composed of IR genes and TFs is helpful to analyze and identify the immune escape pathways adopted by patients in different tumor subtype, and even in a single sample level.

The study conducted by Kaisa Cui et al. showed that the copy number variation (CNV) is a main driving factor for the gene transcription variations related to ribosomal RNA metabolism, rather than SNP (single nucleotide polymorphism) or DNA hypomethylation^12^. Many functionally conserved up-regulated genes are associated with the amplification and deletion of CNV, especially in cancers of the digestive system and respiratory system, such as LUAD. Therefore, we realized that it would be necessary to further study the effects of CNV on LUAD, especially on important IR genes at the population or subtype level.

**Fig. 1:**
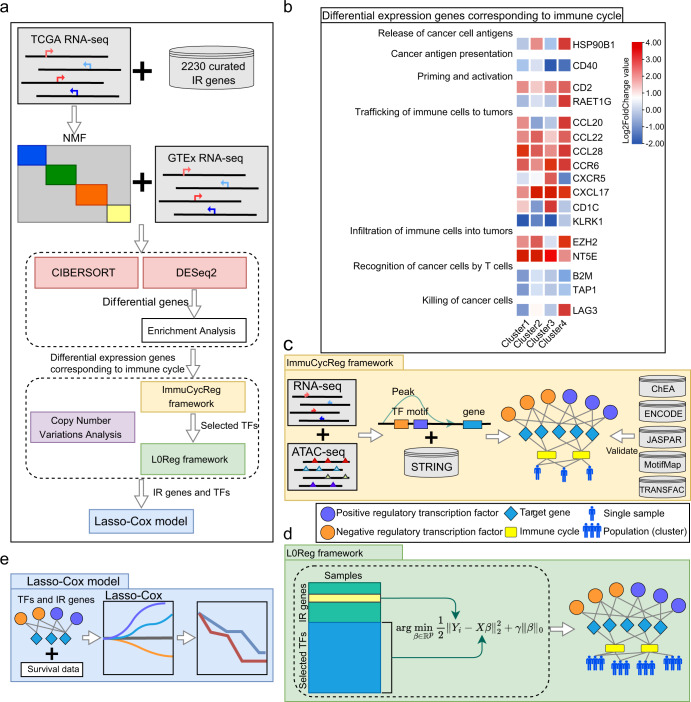
A method for integration of single sample and population analysis. **a** Flowchart of our proposed framework. **b** The differential expression genes corresponding to immune cycle of TCGA-LUAD, red indicates high expression in such clusters, and blue indicates low expression. The expression amount was derived from the results of different analysis, in which *P-*value < 0.05 and Log2FoldChange > 1. **c** The diagram of ImmuCycReg framework. The RNA-seq (TCGA RNA-seq, GTEx RNA-seq) datasets, ATAC-seq dataset and STRING database^67^ were used to construct regulatory network which was validated by the ChEA^56^, ENCODE^55^, JASPAR^54^, MotifMap^57^ and TRANSFAC^58^ databases. **d** The diagram of L0Reg framework. The input data consisted of two parts: an IR gene expression matrix *Y* and a TF encoding gene expression matrix *X*. And, the L0Reg framework output a gene regulatory network on population level. The orange circles are negative regulated TFs, the purple circles represent positive regulated TFs, the blue diamonds denote IR genes, the yellow rectangles denote immune cycle, and the humanoid signs are single sample or population (cluster). **e** For prognosis prediction, a risk scoring model was established on risk factors identified by Lasso-Cox.

At present, immunotherapy based on immune checkpoints has made great progress, but only some patients respond to this kind of treatment^13^, which indicates that immune escape is still the main obstacle to current immunotherapy^14^. In addition, from the perspective of precision medicine, it is necessary to accurately identify the immune defects of patients in order to implement targeted immune enhancement or immune repair. However, at present, there is no effective computational framework that can analyze the immune deficiency of a specific patient from the perspective of a single sample, which hinders the clinical application of personalized medicine. Therefore, in order to systematically identify the possible immune escape pathways, we proposed a computational framework for single sample analysis, and then validated and improved it with the analysis results of population analysis. Firstly, we integrated multi-source (The Cancer Genome Atlas (TCGA), Assay for Transposase-Accessible Chromatin with high throughput sequencing (ATAC-seq) and the Genotype-Tissue Expression (GTEx)) and multi-omics data (genome, transcriptome and proteome), to build a regulatory framework at single sample level for immune cycle analysis of TCGA-LUAD, namely Immune Cycle Regulatory framework (ImmuCycReg framework) (Fig. 1a). Then, we used IR genes as features and clustered TCGA-LUAD samples by non-negative matrix factorization (NMF)^15^, recognizing 4 LUAD immune subtypes. Differentially expressed IR genes between 4 subtypes and GTEx samples were identified by DESeq2^16^, and these IR genes were then aligned to the immune cycle by Gene Ontology (GO) and Kyoto Encyclopedia of Genes and Genomes (KEGG) enrichment analysis. The ImmuCycReg framework can accurately identify TFs that regulate IR genes based on the RNA-seq and ATAC-seq data, and infer the regulatory relationship of TFs to the IR genes, thereby constructing a regulatory network at a single sample level (Fig. 1c). On this basis, we also constructed a population regulatory framework (L0Reg framework) based on L0 regularization technique (Fig. 1d). Combined with the regulatory relationship verified in the ImmuCycReg framework and the immune cell infiltration of these 4 subtypes calculated by CIBERSORT^17^, we analyzed the possible immune escape mechanism at population level. To verify whether the important genes and TFs derived from the L0Reg framework have an impact on the survival, prognosis, and development of LUAD, we calculated prognostic score based on the risk factors identified by Lasso-Cox model (Fig. 1e).

## Results

### The identification of four LUAD subtypes

**Fig. 2:**
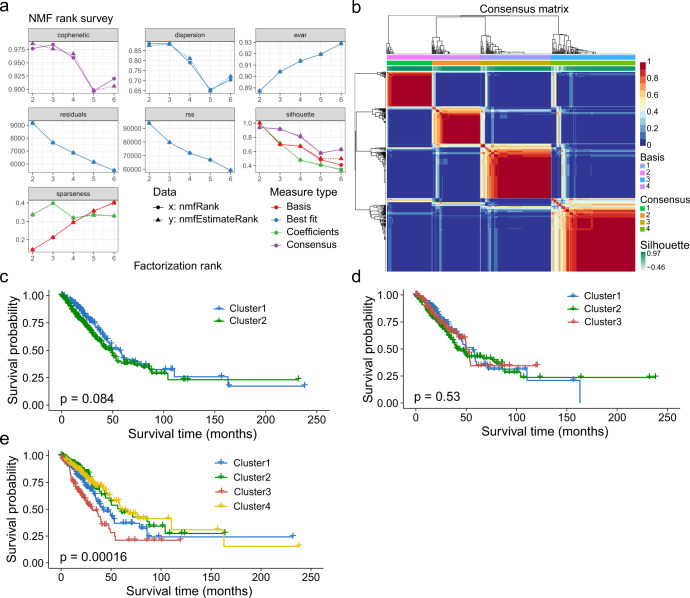
Subtypes stratification and survival analysis on different numbers of clusters. **a** According to the cophenetic parameters of NMF, TCGA-LUAD samples were divided into four subtypes. The line x (solid line) was drawn according to the *nmf* function. The line y (dotted line) was set according to the *nmfEstimateRank* function. These results were plotted to choose the optimal *rank* value. And, the x-axis value is the possible factorization rank (2–6), and the y-axis represents the cophenetic value. **b** Consensus map of NMF clustering results in TCGA-LUAD cohort. **c**, **d** and **e** The OS of patients with the 2, 3 and 4 subtypes were determined by Kaplan–Meier survival analysis, respectively. The *P-*values were calculated by the Log-rank test.

Based on the 2230 IR genes, the NMF approach was utilized to decipher immune subtypes. As illustrated in Fig. 2a, the optimal cluster option was four due to the cophenetic coefficient that started to rapidly decline. The consensus matrices also suggested that four clusters showed optimal stratification (Fig. 2b). Therefore, TCGA-LUAD samples were divided into four subtypes, and the numbers of samples in each subtype were 158, 104, 130 and 100, respectively (Supplementary Data 3 S1). Note that when the number of subtypes is 4, there are significant differences of Overall Survival (OS) (Fig. 2c, d and e), which also indirectly indicates that such clusters are reasonable and have clinical significance.

### Enrichment analysis of immune genes

**Fig. 3:**
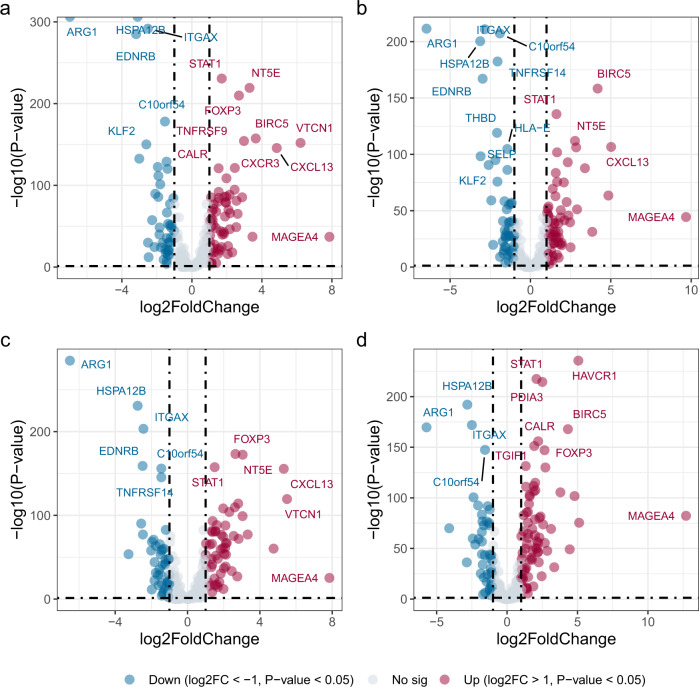
A volcano plot of differential expression genes between four subtypes and GTEx samples. The red dots indicate upregulated genes, blue dots indicate downregulated genes and gray dots are not significantly genes. **a** Cluster 1. **b** Cluster 2. **c** Cluster 3. **d** Cluster 4.

We used DESeq2^16^ to analyze differential expression of 249 immune cycle genes across four immune subtypes and GTEx samples (Fig. 3, Supplementary Data 4), and then we selected 42 of these genes for subsequent analysis (Supplementary Table 1). After preliminary selection of differential expression genes, these 42 genes were input into the ImmuCycReg framework, and finally 17 IR genes showed meaningful results (Supplementary Fig. 1). In addition, we used a heatmap to reveal the expression patterns of these 17 IR genes across the four immune subtypes and GTEx (Supplementary Fig. 2).

**Fig. 4:**
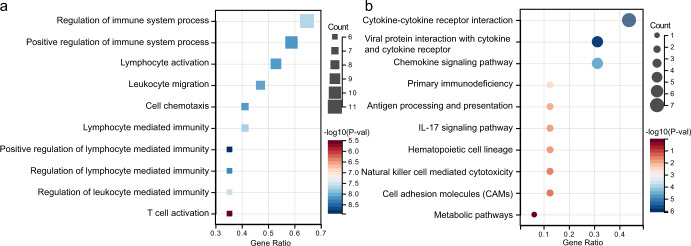
The enrichment analysis of differential expression genes identified by comparing TCGA-LUAD with GTEx samples. **a** GO enrichment analysis. **b** KEGG enrichment analysis.

To discover common transcriptional level changes in LUAD and reveal their influence in functional pathways, we performed GO functional and KEGG pathway enrichment analysis on 17 IR genes by Cytoscape^18^ plug-in ClueGo^19^. We found that the GO functional enrichment terms of these genes were mainly involved in regulation of immune system process, cytokine-mediated signaling pathway, antigen processing and presentation and T cell activation (Fig. 4a). The results of KEGG pathway enrichment analysis were mainly related to cytokine-cytokine receptor interaction, viral protein, interaction with cytokine and cytokine receptor, chemokine signaling pathway, primary immunodeficiency, antigen processing and presentation, IL-17 signaling pathway, hematopoietic cell lineage, metabolic pathways, natural killer (NK) cell mediated cytotoxicity and cell adhesion molecules (CAMs) (Fig. 4b). These results suggested that the function of immune cells in the LUAD patients’ immune microenvironment might be abnormal. According to the results of the enrichment analysis (Supplementary Data 5), and referring to the evidence proposed in other studies on the immune cycle^20,21^, these genes are related to the 7 steps of the immune cycle^20^: release of cancer cell antigens, cancer antigen presentation, priming and activation, trafficking of immune cells to tumors, infiltration of immune cells into tumors, recognition of cancer cells by T cells and Killing of cancer cells. The genes corresponding to different steps in immune cycle were depicted in Fig. 1b and Supplementary Fig. 3.

### The possible immunodeficiency analysis in single sample level

The current immunotherapy based on *PD-1*/*PD-L1* monoclonal antibody has changed the treatment prospects of a variety of cancers, especially suitable for tumors with limited treatment options, such as lung cancer^22^. Studies have shown that these antibodies produce a long-lasting response in some patients without causing serious side effects. However, a large proportion of patients showed innate or acquired resistance to immunotherapy. Before the anti-cancer immune response effectively killing cancer cells, a series of gradual events must be initiated and allowed to continue and iteratively expanded, and these steps are defined as the cancer-immune cycle^20^. The immune escape events are derived from complex cell interactions within the TME during the immune cycle^23^. An important feature of cancer is the accumulation of gene variations and the loss of normal cell regulatory functions^24^, which in turn leads to the changes of interactions between the tumor cells and immune cells and then immune escape events occur.

**Fig. 5:**
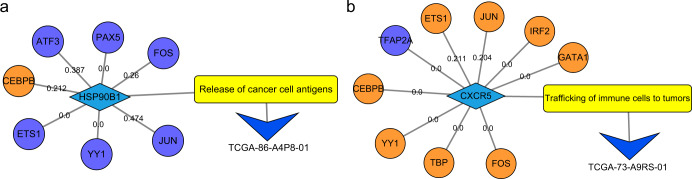
The regulatory network according to the results of the ImmuCycReg framework. The orange circles are negative regulated TFs, the purple circles represent positive regulated TFs, the blue diamonds are IR genes, yellow rectangles are stages of immune cycle, and V-shaped triangles denote single sample. **a** The regulatory network of the *HSP90B1*. **b** The regulatory network of the *CXCR5*.

Through the ImmuCycReg framework, we performed a single sample regulatory analysis of TCGA-LUAD patients to accurately recognize the possible immunodeficiency of each patient. *HSP90B1* is a key gene that promotes antigen release (Fig. 1b). The analysis results showed that due to the regulation of TFs such as ATF3 (interaction score = 0.387), CEBPB (interaction score = 0.212), FOS (interaction score = 0.26) and JUN (interaction score = 0.474) (Supplementary Data 8S2), the low expression of *HSP90B1* might have an impact on the patient TCGA-86-A4P8-01 (Fig. 5a, Supplementary Data 8S1). Note that the interaction scores were directly acquired from the STRING databases and more details are in Methods. It means that this patient may be immunodeficiency at the earliest stage of the immune cycle. We also found that the CEBPB is a negative regulatory TF of *HSP90B1*, and inhibits its expression^25^.

The significantly lower expression of *CD1C* and *CXCR5* may affect T cell recruitment, and we found that the tumor antigen presentation process of patient TCGA-73-A9RS-01 might be deficient (Supplementary Data 8S1), resulting in the inability to detect tumor antigens. Consequently, dendritic cells (DCs) and T cells cannot use antigens as their immune response, thereby failing to recognize cancer cells, and then Treg cells emerge, promoting tumor development^26^. Through regulatory analysis, we found that the antigen presentation function of patient TCGA-73-A9RS-01 might be influenced by ETS1 (interaction score: 0.211), JUN (interaction score: 0.204) and other TFs on *CXCR5* (Fig. 5b, Supplementary Data 8S2).

When different lymphocytes infiltrated into the TME, they will be involved in the tumor immune response of the primary tumor and metastasis sites^27^, and some of these responses can lead to the occurrence of tumor immune escape. It has been proved that the significantly high expression of *EZH2* can promote the proliferation of colon cancer cells^28^. We found that *EZH2* was highly expressed in 7 samples (Supplementary Data 8S1). Through regulatory analysis, we found that YY1 (interaction score = 0.999), JUN (interaction score = 0.783), RXRA (interaction score = 0.781), TBP (interaction score = 0.379), CEBPB (interaction score = 0.337), TFAP2A (interaction score = 0.195), and other TFs had positive regulatory effects on *EZH2* (Supplementary Fig. 4a, Supplementary Data 8S2), probably promoting immune escape. The high expression of YY1 can promote the transcription of multiple immunosuppressive genes^29^, thereby promoting the growth of TCGA-LUAD. And, the TFAP2A can greatly activate the signal that promotes epithelial-mesenchymal transition (EMT) in LUAD, so that epithelial tumor cells undergo morphological remodeling, cell adhesion reduction, and enhanced migration and invasion capabilities^30^. It means that the TFAP2A may result in metastasis which is the main cause of cancer-related death^31^. In this study, we also found that the TFAP2A showed high correlation with the poor prognosis of LUAD (Fig. 8c).

The *LAG-3*, an immunosuppressive receptor, mainly presents on activated T cells^32,33^, and its ligand is major histocompatibility complex class II (MHC-II). The *LAG-3* is mainly used as a receptor for transmitting inhibitory signals^34^, and negatively regulates the proliferation, activation, effector function, and homeostasis of CD8 + and CD4 + T cells. In addition, the *LAG-3* expresses on some regulatory T cells and contributes to their inhibitory function^35^. In this study, we found that the *LAG-3* highly expressed in eight samples under the regulation of STAT4 (interaction score = 0.321, Supplementary Data 8S2). Chen et al. found that the *FGL1* is the main ligand of *LAG-3*^36^, which shows association with poor prognosis and treatment outcomes of LUAD. By blocking the *FGL1*/*LAG3* interaction, antitumor immunity can be promoted by stimulating tumor-infiltrating lymphocyte activation and expansion in the TME^36^.

### The possible immunodeficiency analysis in population level

**Fig. 6:**
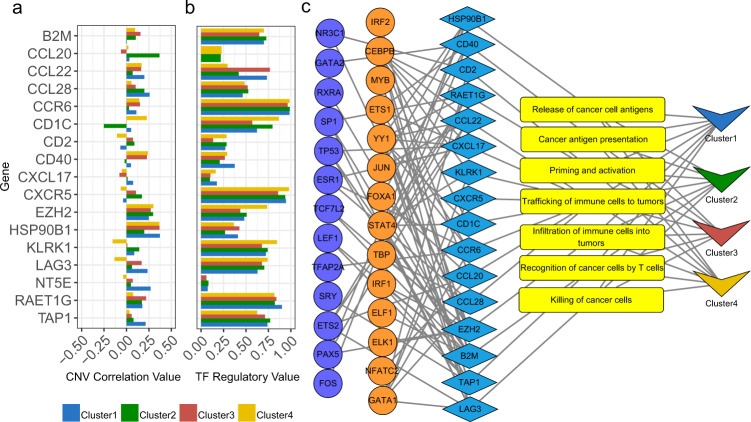
The CNV correlation and regulatory relationship results of four clusters. **a** The extent to which IR genes are influenced by CNV in the L0Reg framework. **b** IR genes are regulated by the TFs identified by the L0Reg framework. **c** The regulatory network drawn based on the results of the L0Reg framework. Red circles are negative regulated TFs, blue circles denote positive regulated TFs, blue diamonds are IR genes, yellow rectangles are stages of immune cycle, and V-shaped triangles represent clusters.

We also analyzed these 4 immune subtypes by the L0Reg framework (Fig. 1d) in population level. The L0Reg framework can simultaneously analyze the correlation between CNV and IR gene expression (*Spearman* correlation coefficient) and the regulatory effect of TFs on IR gene. For example, in Cluster 1, 3, and 4, the *Spearman* correlation between the expression of *HSP90B1* and CNV all reached 0.36 (Fig. 6a), which meant that the low expression of *HSP90B1* might be related to CNV. And, the low expression of *HSP90B1* may inhibit antigen release of cancer cell, which causes immune cells failing to recognize tumor cells (Fig. 1b). In another important stage of the immune cycle, the results showed that the *CD1C* might play an important role in the recruitment of T cells (Fig. 1b), and *CD1C* was not significantly expressed in Cluster 2 and 4 (Supplementary Table 1). And then, we constructed a regulatory network of the *CD1C* by the L0Reg framework (Fig. 6c). Here, we used R-squared (*R*^2^) denote a TF regulatory value which is the proportion of samples that may be influenced by these TFs in each subtype (see Methods for more details). In Cluster 2 and 4, the TF regulatory values were 0.79 and 0.86, respectively (Fig. 6b, Supplementary Data 8S4), which indicated that the low expression of *CD1C* might be caused by the regulatory effect of TFs.

### The differential expression and regulatory analysis of Chemokines

Chemokines play a vital role in guiding the migration of immune cells which are necessary to initiate and transmit an effective anti-tumor immune response. Chemokines and their respective receptors are considered to be the key regulators of the tumor vasculature and have a dual role in tumor angiogenesis^27^. The secretion of chemokines in TME usually changes. Abnormal chemokines promote the differentiation and infiltration of immunosuppressive tumor-promoting cells into tumors. The *CXCL17* is highly expressed in various cancer cells and induces the expression of vascular endothelial growth factor (VEGF) in monocytes and endothelial cells^37,38^, and promotes tumor growth by increasing angiogenesis and enhancing immune escape^37,39^. In this study, we also found that the *CXCL17* showed higher expression level in all clusters than GTEx (Fig. 1b and Supplementary Table 1), and it interacted with other molecules in TME and promoted tumor growth and enhanced immune escape^37,39^. For example, in the Cluster 1, the results of L0Reg framework showed that the TP53 (*P*-value = 0.00062) and YY1 (*P*-value = 0.001593) positively regulated the expression level of *CXCL17*. In the Cluster 2, the IKZF1 (*P*-value = 0.001154) and IRF1 (*P*-value = 9.72e-11) positively regulated the expression level of *CXCL17*, while the STAT1 (*P*-value = 0.006027) negatively regulated the expression level of *CXCL17*. The results of Cluster 3 and Cluster 4 were supplemented in Supplementary Data 8S3. Interestingly, previous studies also proved that different TFs can confer latent plasticity to the *CXCL17* to perform different functions in different cell types and conditions^11^. Therefore, it should be noted that the high expression of *CXCL17* may also trigger an immune response^40^, allowing DCs, CD8 + , and CD4 + T cells to be recruited into the tumor and suppressing cancer progression. For example, in our study, the L0Reg framework found that the *CXCL17* was regulated by YY1 (*P*-value = 0.003152), CEBPB (*P*-value = 0.014052) and ESR1 (*P*-value = 0.010408) in Cluster 4 where the high expression level of *CXCL17* showed a better prognosis (Figs. 6c and 8d, Supplementary Data 8S3).

**Fig. 7:**
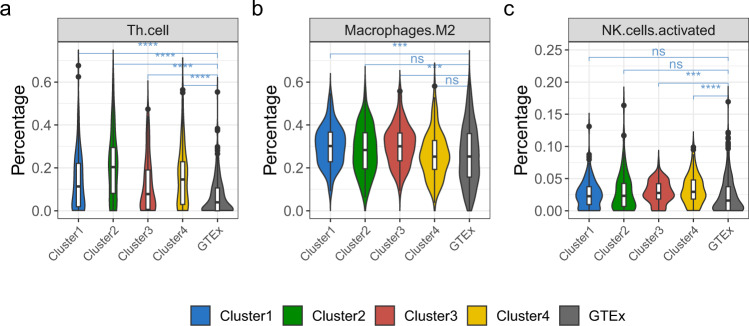
Comparison of Th cell, M2, and activated NK cell between four clusters and GTEx. The differences between groups were measured by the Kruskal Wallis test. * Indicates *P*-value < 0.05, ** Indicates *P*-value < 0.01, *** Indicates *P*-value < 0.001, **** Indicates *P*-value < 0.0001.

T helper (Th) cell is a common immune cell subgroup in TME. Studies showed that Th cells could be recruited by the *CCL20*-*CCR6* axis and then migrate into the TME, promoting tumor development^26^. Through the L0Reg framework, we found that the *CCL20*-*CCR6* axis enriched in Cluster 2 and 4 and it was regulated by multiple TFs (Fig. 6c), and the CEBPB (*P*-value = 0.000417) was a major TF (Supplementary Data 8S3). We used CIBERSORT^17^ to assess the infiltration degree of Th cells, and we found that the proportion of Th cells was significantly higher in the four subtypes of TCGA-LUAD than GTEx samples (Fig. 7a). The results of immune infiltration analysis are consistent with the results of regulatory network analysis on population level.

### Effect of tumor-associated macrophages on TCGA-LUAD

In the TME of LUAD, a large number of tumor-associated macrophages (TAMs) are associated with lymphangiogenesis and lymphatic metastasis^41^. TAMs are considered to differentiate into two main phenotypes: pro-inflammatory (M1) and tumorigenic (M2). The M2 type macrophages cells have strong immunosuppressive properties, and a large amount of M2 is also an independent factor leading to poor prognosis. Like Treg cells, the M2 also inhibits the activation of tumor-associated antigen (TAA)-specific CD8 + T cells. And, we found that the proportion of M2 in Cluster 1 and Cluster 3 were significantly higher than the normal sample (Fig. 7b). Previous studies proved that M2 would be recruited into the TME by the *CCL22*^42^. Then, we analyzed the regulatory relationship of the *CCL22*. According to the regulatory network of the *CCL22* derived by the L0Reg framework, we found that in Cluster 1 and Cluster 3, the TF regulatory values are 0.73 and 0.76, respectively (Fig. 6b). It means that the high expression of *CCL22* in Cluster 1 and Cluster 3 may be due to the regulation of TFs (Supplementary Data 8S4), thereby promoting the enrichment of M2. For example, the regulatory relationship between TFs and *CCL22* of TCGA-49-AARO-01 and TCGA-MP-A4T4-01 was detailed in Supplementary Data 8S1.

### Prognosis scoring based on the Lasso-Cox model

**Fig. 8:**
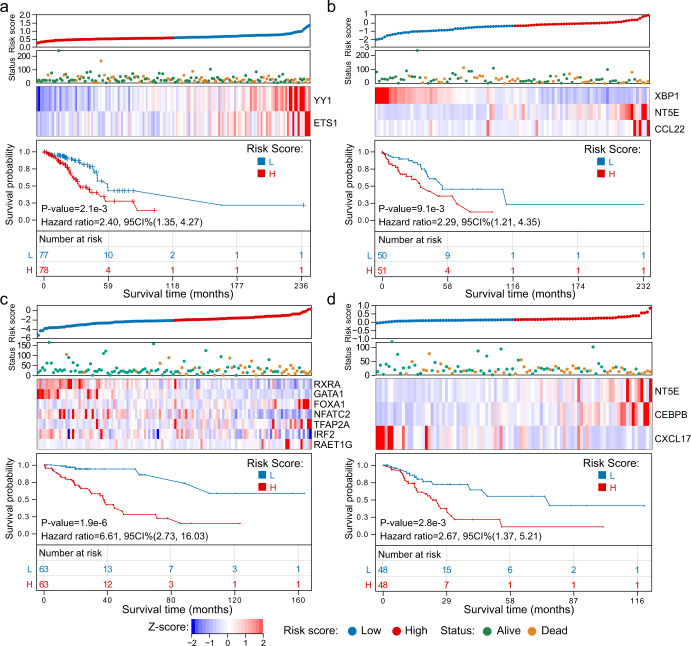
Lasso-Cox prognostic risk scoring models. **a**, **b**, **c** and **d** are the results of Lasso-Cox prognostic risk scoring models of Cluster 1, 2, 3 and 4, respectively. According to the Risk Score, the samples were divided into high-Risk Score group and low-Risk Score group 1:1. The Kaplan–Meier curve showed the relationship between Risk Score and survival.

Through the analysis results of the ImmuCycReg framework and L0Reg framework, the regulatory relationships between IR genes and TFs have been derived. To verify whether these genes and TFs play an important role in the development and prognosis of LUAD, we constructed a prognostic risk scoring model based on the Lasso-Cox (Fig. 8a–d). And according to the prognostic Risk Score of the patients in various clusters (Supplementary Data 9), the patients were then divided into a low-Risk Score group and a high-Risk Score group. As the expression level of cancer-promoting IR genes or TFs increases, the patient’s Risk Score increases and OS gets worse. The Kaplan–Meier curve showed that patients with high-Risk Score might have poor OS^43^. The time-dependent area under curve (AUC) showed that risk features in various clusters could be effective OS indicators (Supplementary Fig. 6a–d and Supplementary Fig. 7a). Then, according to the risk features in various clusters, we used *R* package *rms* to depict the prognostic nomogram of various clusters (Supplementary Fig. 7b–e).

The results of prognostic model on the Cluster 3 showed that the high expression of the *RAET1G* would be a sign of poor prognosis of LUAD (Fig. 8c). Note that previous studies also have shown that the *RAET1G* plays important role in a variety of tumors (e.g. lung cancer)^44^ and it downregulates the expression of *NKG2D* (also known as *KLRK1*) on NK cells^45^. Consequently, the low expression of *NKG2D* will weaken the ability to mediate the cytotoxicity of NK cells^45^. This may partly explain why the proportion of activated NK cells in Cluster 3 and 4 are higher than that normal group (Fig. 7c), but there is no better prognostic performance. The *RAET1G* is expressed in a series of different epithelial tumor types and the role of *RAET1G* in tumor progression deserves further study^44^.

## Discussion

The formed tumors are heterogeneous and complex, composed of malignant proliferating cells and undifferentiated cell components, including mesenchymal cells, blood vessels, and inflammatory cells^42,46,47^. A large amount of existing evidence showed that TME contains a variety of adaptive immune cells such as T lymphocytes and B lymphocytes, as well as innate immune cells such as macrophages, DCs, polymorphonuclear neutrophils (PMNs), NK cells, eosinophils, mast cells and Myeloid-derived suppressor cells (MDSCs)^48^. These different types of immune cells functionally interact with tumor cells, which may promote tumor progression and metastasis^47^. In this study, we proposed the ImmuCycReg framework and the L0Reg framework for integrating multivariate, multi-source and multi-omics data. Identifying the immune deficiency of a single sample is a prerequisite for applying immunotherapy to personalized medicine. The regulatory network frameworks constructed in this study is analyzed at the single sample level, and then combined with the stratification of LUAD subtypes to carry out population level analysis, so as to find the potential escape pathways from a system perspective. Note that for population analysis, we proposed the L0 regularized regression model for inferring a dependent variable (the expression level of IR gene) and multiple independent variables (candidate TFs inferred by the ImmuCycReg framework). Our regulatory network inference approach has several advantages. Firstly, the L0 regularization technique cannot only accurately identify the TFs most related to the IR genes, but also eliminate irrelevant TFs without manually setting a threshold. Secondly, the L0Reg framework can recognize the regulatory effect between TF and target gene both activating and inhibiting. Thirdly, the credible regulatory relationship derived by the ImmuCycReg framework can be inputted into the L0Reg framework to improve the computational efficiency of the L0Reg framework and avoid false positive results as much as possible. Finally, compared with the elastic network regression model^10^, the L0 regularization technique has better performance in processing high-dimensional sparse data^49^ and the L0Reg framework outperforms GENIE3^7^ in the overall performance of reconstructing regulatory networks (Supplementary Fig. 5). In addition, our regulatory framework has satisfactory transfer ability, which has potential value in pan-cancer analysis, even in single cell RNA-seq data analysis.

The experimental results showed that our proposed framework could recognize tumor progress and escape related pathways adopted by different subtypes or even individual patient. For example, the low expression of *HSP90B1* may inhibit antigen release of cancer cell, which causes immune cells failing to recognize tumor cells; The *CXCL17* is highly expressed in all clusters and may induce the expression of VEGF, promoting tumor growth by increasing angiogenesis; the Th cells are recruited by the *CCL20*-*CCR6* axis and then migrate into the TME, thereby resulting in the formation of immunosuppressive TME.

Our research also revealed the impact of IR genes on the clinical significance of different subtypes of LUAD. And, we used the Lasso-Cox to establish a prognostic risk scoring model to predict the survival and prognosis of LUAD patients. Our results showed that the Risk Score could be an independent prognostic factor and have a considerable impact on predicting OS of LUAD patients. We have also noticed that the high expression level of *CD73* (also known as *NT5E*) in LUAD is significantly correlated with poor OS (Fig. 8b, d), which may indicate that *CD73* plays an indistinct role in tumor development. Previous studies proved that the *RAET1G* might downregulate the expression level of *NKG2D* on NK cells, thereby evading immune recognition^45^. However, some oncogenes that are of great significance but not significantly expressed, such as *RAET1G*, cannot be identified by traditional univariate analysis method. In this study, based on the results of the integration analysis of single sample level and population level, we believed that the function of *RAET1G* deserves further study.

There are still some shortcomings in our research. First of all, although we used multi-source and multi-omics data, our main basis was still derived from transcriptome. However, the transcriptome analysis can only reflect some aspects of immune status. For example, there may be other important immune escape pathways involved in the patient’s immune cycle, but it needs comprehensive analysis by systematic approach^50^. Secondly, although the TCGA collected a large number of patients with good follow-up data, it was based on bulk sequencing technology, which meant that the signal of each patient was the average signal of all cells in tumor tissue. In other words, the signals of many important tumor subclones and immune cells in different states were averaged, which masked the details of the interaction between tumor cells and immune cells to a certain extent. Finally, our results need to be verified by further experiments in vivo and in vitro.

## Materials and methods

### Multi-source and multi-omics datasets

Wang et al.^51^ processed the LUAD samples in TCGA and GTEx^52^ to eliminate batch effects. Note that the batch effect of the preprocessed data with 503 TCGA-LUAD samples and 313 GTEx normal samples have been properly eliminated. In this study, the TCGA and GTEx data were quantile normalized using the R scale function to follow the average empirical distribution observed across samples. Therefore, we used preprocessed data in high quality, including 492 LUAD samples (Supplementary Data 3 S1) and 313 GTEx normal samples (Supplementary Data 3S2). Note that only 478 of these 492 patients were accompanied by complete diagnostic data, so that only these 478 samples (Supplementary Data 3S3) were used in the construction of the Lasso-cox model. The ATAC-seq data was downloaded from UCSC- Xena^53^ (https://atacseq.xenahubs.net), and there were 20 LUAD samples.

The JASPAR^54^, ENCODE^55^, ChEA^56^, MotifMap^57^, and TRANSFAC^58^ databases provided the currently known TFs’ regulatory relationship to gene, and in this study, these databases were used to validate the regulatory relationship recognized by the ImmuCycReg framework.

### Collecting candidate immune-related genes

To understand how these tumor cells evade damage from immune cells (such as T cells, NK cells, and so on), we should first carry out comparison of candidate immune-related genes between tumor and normal tissues. Patel et al. conducted a comprehensive investigation on essential genes for cancer immunotherapy and provided gene-ranking based on small guide RNA (sgRNA) enrichment analysis^59^. In addition, we also manually collected some important NK ligands. Therefore, we collected a total of 2230 candidate immune-related genes (IR genes) (Supplementary Data 1).

To identify the signature genes that influence the TCGA-LUAD immune cycle^20^, we also selected 249 stimulatory and inhibitory genes^60^ (178 genes were reported by Liwen Xu et al.^21^ and other 71 genes were collected from other studies^20,61–63^). These genes involved in the seven steps of the cancer-immunity cycle shown in Supplementary Data 2.

### Non-negative matrix factorization for clustering

To identify tumor subtypes with potential differences in immune escape pathways, the NMF was performed to cluster these TCGA-LUAD samples on 2230 IR genes. In order to make the result of subtype stratification more reliable, we set seed = 20210101 and used the *nmfEstimateRank* function to test different number of iterations (10–50) to determine the most suitable number of iterations. Finally, when the number of iterations was 50, we got a robust estimate of the factorization *rank*. Ran *nmf* function according to these parameter settings (seed = 20210101, number of iterations = 50, possible factorization rank = 2–6, other parameters default). Cophenetic coefficient was employed to determine the optimal *rank*, and silhouette statistic was used to quantify the robustness of clustering patterns. Usually, when the value of cophenetic correlation coefficient starts decreasing, it is deemed as the optimal factorization *rank*^15^.

### DESeq2 for gene expression differences analysis

We used DESeq2^16^ to analyze gene expression differences between these 4 immune subtypes and GTEx samples, and selected IR genes with *P-*value < 0.05 and Log2FoldChange > 1. If a gene is a stimulatory gene and is not significantly highly expressed in any cluster, it will be included in the differential gene set, and if the gene is an inhibitory gene and is significantly highly expressed in any cluster, it will be included in the differential gene set.

### CIBERSORT for immune infiltration analysis

To reveal the distinction of immune cell infiltration levels between these 4 clusters and GTEx samples, we calculated the fraction scores of 36 types of immune cell using CIBERSORT^17^. The 36 types of the immune cell, including 14 types from Miao et al.^64^. and 22 types from CIBERSORT^17^. Note that the input of CIBERSORT is the transcripts per million (TPM) data.

### GO and KEGG enrichment analysis

The GO and KEGG enrichment analysis were carried out by Cytoscape 3.8.2^18^ plug-in ClueGo^19^. Note that the parameters were set as: *P-*value cutoff = 0.05, Correction Method Used = Bonferroni step down, Min GO Level = 0 and Max GO Level = 8. The results of GO enrichment analysis included 3 categories: molecular function (MF), cellular component (CC) and biological process (BP).

### The *Spearman* correlation for copy number variations analysis

The CNV is an important factor causing gene expression variation, and the impact of CNV on the expression variation of cancer-related genes can be quantified by the *Spearman* correlation coefficient^65^. In this study, we also used the *Spearman* correlation in the regulatory framework to calculate the correlation degree between IR genes and CNVs, that is, the CNV correlation value (CNV correlation value ∈ [−1,1]). The higher the absolute value, the greater the influence of CNV on IR gene expression level.

### Immune cycle regulatory framework for single sample analysis

The key step in constructing the ImmuCycReg framework is to identify which TFs regulate the target IR gene. For the peak signal provided in the ATAC-seq data, the peak signal within 20 kb from the Transcription Start Sites (TSS) site of the target gene is usually considered to be TF binding region. Since there might be lots of candidate peak signals within 20 kb of the target IR gene, the *Spearman* correlation coefficient was applied to measure the correlation coefficient between the expression level of the IR gene and the peak signal. The significant correlation coefficient represents that the TFs in the peak region may regulate the expression level of the target IR gene. There were 23,729 peaks in ATAC-seq data of TCGA-LUAD samples and we selected the peak with the highest correlation coefficient to IR gene with *P*-value < 0.01 (Supplementary Fig. 1). The peak DNA sequences were obtained by bedtools^13^, and the TFs were obtained by the online tool PROMO^14^, and then these TFs were used as input for the downstream analysis (Supplementary Data 6). Consequently, a set of TFs for each IR gene was derived. To determine whether the IR gene expression level was up- or downregulated in the single sample level analysis, we performed the Student’s *t*-test statistical analysis^66^ between the TCGA-LUAD samples and GTEx normal samples. Note that the open chromatin is a prerequisite for the binding of TFs, which means that whether it is positive or negative regulation, the chromosome accessibility should be high, that is, the peak signal value should be high. When the peak signal is high and the gene encoding the TF is also highly expressed, if the TF positively regulated the IR gene, the expression level of the IR gene should be also high. Instead, if the peak signal is high and the IR gene is lowly expressed, then the IR gene may be negatively regulated by repressors. Because it is difficult for a single sample to carry out statistical analysis and test the reliability of the results, we integrated the STRING database^67^ into the regulatory framework to verify the regulatory relationship, and at the same time acquired the interaction score from STRING to weight each edge in the regulatory network. And we compared the regulatory relationship speculated by the ImmuCycReg framework with the widely used TF-gene databases, such as JASPAR^54^, ENCODE^55^, ChEA^56^, MotifMap^57^, and TRANSFAC^58^ (Supplementary Fig. 4b). In addition, we also used the statistical method (Student’s *t*-test, *P*-value < 0.001) to identify whether the expression level of the IR gene on a single sample is higher than its expression level of GTEx samples. In this way, it is possible to reveal the regulatory relationships of the IR genes for each sample, individually.

### L0 Regulatory framework for population level analysis

We proposed the L0Reg framework to analyze the immune escape pathways of different TCGA-LUAD subtypes from a population perspective. With using the L0Reg framework, we can further analyze the regulatory relationship between IR genes and selected TFs (high-interaction score derived from the ImmuCycReg framework) on population level. Assuming that the gene expression profile data was properly normalized in advance, therefore the distribution of each gene and TF in all samples approximately followed a standard normal distribution^10^.

The L0Reg framework decomposes the network inference problem into IR genes regression problem which aims to predict the expression values of a target IR gene using the selected TFs. The general principle of the network inference is depicted in Fig. 1d. The L0Reg framework provides a ranking of the regulators of the IR gene according to a weight vector derived from regularization regression models, such as L0 regularization. And we used the absolute value of the fitted feature coefficients as regulatory strength between TFs and genes in population level analysis. The feature selection technique used here is thus supposed to be able to calculate feature importance of each TF to IR gene. In principle, any feature selection technique dealing with a continuous output and returning some kind of feature importance score can be used in our framework.

Hazimeh et al. proposed a method based on coordinate descent and local combinatorial optimization to effectively solve the L0 regularization problem^49^, and developed an *R* package *L0Learn*^49^. With using the *L0Learn*, we designed a TF selection model based on L0 regularization, as shown in Formula (1). In this study, the original gene expression matrix was divided into an IR gene expression matrix *Y* ($Y \in \mathbb{R}^{n \times m}$ with *n* samples and *m* IR genes) and a gene expression matrix *X* of encoding TF ($X \in \mathbb{R}^{n \times p}$ with *n* samples and *p* TFs).

1 $$\arg \mathop {{min}}\limits_{\beta \in \mathbb{R}^p} \frac{1}{2}\left\| {Y_i - X\beta } \right\|_2^2 + \gamma \left\| {\beta _0} \right\|$$

where *Y*_*i*_ is the expression level of the *i*-th IR gene, and *X* is the matrix of TFs, *β* is the regression coefficient vector of TFs, and *γ* controls the number of TFs with non-zero coefficients. Suppose that there are *m* IR genes, so that we could separately use the Formula (1) *m* times to identify different TFs sets related to each target IR gene.

After selecting key TFs through the L0 regularization model, summary statistics are used to replace the original data to improve the calculation efficiency and transfer ability of the model to a wide range of data types^68^. The L0 regularization model can automatically select feature TFs that may regulate the expression level of the IR gene. According to the expression level of each IR gene and TFs in all samples, the *P-*value was obtained to reflect the statistical relationship between them (Supplementary Data 8S3). It should be noted that the multivariate regression model can analyze the effects of multiple TFs on one IR gene at the same time, which is different from the univariate model considering the effect of only one TF. Here, we calculated the TF regulatory value by *R*-squared^69^ (*R*^2^ ∈ [0,1]) to represent the proportion of samples that may be influenced by the TFs identified by the L0Reg framework in each subtype.

### Lasso-Cox prognosis scoring model

The Lasso has been widely used for regularization and dimensionality reduction tasks, which also can be combined with the Cox model^70^ for biomarker screening for survival analysis. However, using the Lasso-Cox model to calculate the prognostic analysis scores of a large number of genes and TFs requires a lot of computational cost and also results in false positives. In this study, in order to narrow down the combination space to be searched and reduce false positives, we performed an unbiased search on the combination of the IR genes and TFs generated by the L0Reg framework. To find out which risk factors are significantly associated with patients’ survival data, the survival analysis and the Lasso-Cox risk analysis were carried on 478 TCGA-LUAD patients with complete clinical information and the numbers of patients in various clusters were 155, 101, 126 and 96, separately. The TCGA-LUAD dataset was divided into training and validation sets in a 7:3 ratio using the *R* package *glmnet*^71^. Therefore, we used the *R* package *glmnet*^71^ to implement the Lasso-Cox model to describe the relationship between dependent variables (survival time, survival status) and independent variables (TF and IR genes), and then feature TFs and IR genes were identified. The Lasso-Cox analysis was carried out with prognostically relevant IR genes and TFs to obtain coefficients and hazard ratios as shown in Supplementary Fig. 7a. It should be noted that in the Lasso-Cox analysis, we only considered the patient’s survival time, survival status, IR genes and TFs, but gender, age, cancer stage and other variables were excluded. In addition, we set up 10-fold cross-validation to obtain the optimal model. The Risk Score is defined as Formula (2).

2 $${{{\mathrm{Risk}}}}\;{{{\mathrm{Score}}}} = {\sum} {\zeta _i \ast Exp_i}$$

where *ζ*_*i*_ is the coefficient of the gene or TF in the Lasso-Cox model, and *Exp*_*i*_ is the expression value of the gene or TF. The final risk models are:

The Risk Score of Cluster 1 = 2.1940e-4 * ETS1 + 3.1814e-4 * YY1

The Risk Score of Cluster 2 = −9.1679e-5 * XBP1 + 2.2958e-4* *NT5E* + 8.1015e-4* *CCL22*

The Risk Score of Cluster 3 = −2.039e-3 * RXRA − 6.2479e-2 * GATA1 + 2.9037e-4 * FOXA1 − 6.0539e-3 * NFATC2 + 1.0598e-3 * TFAP2A − 3.6931e-4 * IRF2 + 2.1020e-3 * *RAET1G*

The Risk Score of Cluster 4 = 1.8979e-4 * *NT5E* + 5.4765e-5 * CEBPB - 3.6880e-6 * *CXCL17*

Finally, the time-dependent area under curve (AUC) was plotted by the *R* package *timeROC*^72^, the survival curves were plotted using the *R* package *survival* and the prognostic nomograms were depicted by using the *R* package *rms*.

### Reporting summary

Further information on research design is available in the Nature Research Reporting Summary linked to this article.

## Supplementary information

Supplementary Information

Reporting Summary

Supplementary Data 1

Supplementary Data 2

Supplementary Data 3

Supplementary Data 4

Supplementary Data 5

Supplementary Data 6

Supplementary Data 7

Supplementary Data 8

Supplementary Data 9

## Data Availability

Publicly available datasets were analyzed in this study. Processed data are available in the reproducibility GitHub repository: https://github.com/mengxu98/ImmuCycReg-framework/tree/main/data. Results of the regulatory networks obtained by the ImmuCycReg framework and the L0Reg framework were detailed in Supplementary Data 8.

## Code availability

Implementations of the ImmuCycReg framework and L0Reg framework in *R*, with appropriate documentation, are available at GitHub repository: https://github.com/mengxu98/ImmuCycReg-framework.
