# Supplementary material for: Integration of single sample and population analysis for understanding immune evasion mechanisms of lung cancer
Source: NPJ Syst Biol Appl. 2023 Feb 10;9:4. doi: 10.1038/s41540-023-00267-8 (PMC9918494; doi:10.1038/s41540-023-00267-8)
Supplement: Supplementary file 1 — Supplementary Information [file 41540_2023_267_MOESM1_ESM.pdf]

Supplementary Information for "Integration of single sample and population analysis for understanding Immune evasion mechanisms of lung cancer"

Xiong Li[1,*], Xu Meng[1], Haowen Chen[2], Xiangzheng Fu[2], Peng Wang[2], Xia Chen[2], Changlong Gu[2] and Juan Zhou[1]

[1]School of Software, East China Jiaotong University, Nanchang, 330013, China

[2]College of Computer Science and Electronic Engineering, Hunan University, Changsha, China

***Corresponding author: Xiong Li, lx_hncs@163.com**

Supplementary Materials

The results of simple and traditional transcriptomic analysis

The tumor heterogeneity means that different tumor subtypes may adopt different immune escape pathways, so that different subtypes may show special gene expression patterns. Therefore, the non-negative matrix factorization (NMF)[1] clustering results of TCGA-LUAD subtypes were compared with the GTEx samples for differential expression analysis to identify IR genes that play key roles in tumor development and evolution. In this study, the differential expression IR genes were identified by DESeq2[2].

The immune genes with P-value < 0.05 and Log2FoldChange > 1 will be further analyzed. If a gene is a stimulatory gene and is not significantly highly expressed in any cluster, it will be included in the differential gene set. And, if a gene is an inhibitory gene and it highly expressed in any cluster, it will be included in the differential gene set. Therefore, 42 genes were derived and detailed in Supplementary Table 1.

The heatmap was used to visualize specific features of clusters, as shown in Supplementary Figure 2. However, the heatmap cannot reveal the interactions between genes. Co-expression clusters of similar transcriptional responses obtained by clustering algorithms may provide a rough co-expression relationship between genes through molecular network. However, the causal regulatory relationship between genes cannot be determined by such network. The main purpose of constructing a gene regulatory network (GRN) is to find out how transcription factors (TFs) regulate the expression level of target gene. The nodes of the GRN are genes, and the edges represent the interactions between genes and TFs. In a GRN, direct interactions between genes represent their causal regulatory relationships. In order to reliably reveal dynamic biological processes, methods for constructing GRNs are emerging. With using Pavlidis Template Matching (PTM)[3] and GENIE3[4] methods, Potier, D. et al. constructed a GRN during eye development in Drosophila[5]. The PTM is a feature selection method for building GRNs based on analysis of variance (ANOVA) and "Template Matching". They calculated the correlation degree

between genes and candidate transcription factors (TFs) by *Pearson* coefficient, and the TFs with significant correlation value were considered as the regulatory TFs of the target gene. However, it could not describe the possible epistatic effect of multiple TFs, and the ANOVA might be unstable. The GENIE3 selects features by regression tree model and then infers GRN with using repeated random subsampling technique. However, the GENIE3 cannot construct regulatory network on single sample level, which means that the immune escape pathways adopted by a specific patient cannot be determined for personalized precision medicine.

Therefore, we proposed the Immune Cycle Regulatory (ImmuCycReg) framework for single sample level regulatory analysis and the L0 Regulatory (L0Reg) framework for population level regulatory analysis.

Cross-validation and fitting accuracy of L0Reg framework

We evaluated the performance of our L0Reg framework by the root mean square deviation (RMSD) and the *Pearson* correlation (R) between prediction and real expression level[6]. We conducted three kinds of experiments to estimate the performance of our model under control conditions:

(1) We input a random dataset of the same size as the TCGA-LUAD dataset, and the random dataset was normalized and preprocessed in the same way, and then the model was trained and tested by 10-fold cross-validation. The average $RMSD_{test} = 1.0062$ and $R_{test} = -0.04$ (Supplementary Data 7 S1). (2) The expression profile data of TCGA-LUAD was randomly shuffled for 10-fold cross-validation. The average $RMSD_{test} = 1.0546$ and $R_{test} = 0.0006$ (Supplementary Data 7 S2). (3) For the TCGA-LUAD dataset, the average $RMSD_{test} = 0.0329$, $R_{test} = 0.86$ (Supplementary Data 7 S3).

These results suggested that the regulatory relationships between TFs and IR genes might be unlikely to occur randomly. And, the L0Reg framework showed the satisfactory ability to predict gene expression level through TFs, and it could be also feasible to identify the TFs of target IR gene.

Performance comparison of L0Reg framework and GENIE3

We evaluated the L0Reg framework and GENIE3 on 5 gene expression profile datasets provided by the Dialogue for Reverse Engineering Assessments and Methods 4 (DREAM4) challenge[7]. In order to compare them more objectively, we also simulated 10 gene expression datasets by the GeneNetWeaver (GNW-100) [8,9]. Supplementary Table 2 listed the details of all these datasets.

The overall top performer of the DREAM4 challenge was the GENIE3 algorithm which solved the network inference problem by decomposing it into a separate regression problem for each target gene. The GENIE3 used a tree-based ensemble method to calculate a feature importance for weighting the regulatory relationship between the predictor and the target gene in the GRN. In the L0Reg framework, we used the absolute value of the fitted predictor coefficients as feature importance. We compared the performance of the L0Reg framework and GENIE3 based on the ranking of GRN edges, and all edges were sorted in descending order of confidence, and only the top 100,000 prediction edges were kept.

Finally, we calculated the Area Under the Receiver Operating Characteristic curve (AUROC) according to the gold standard of regulatory relationship provided by DREAM4. We ran the L0Reg framework and GENIE3 10 times on each dataset and calculated the AUROC, taking the average of all the results (Supplementary Figure 5, Supplementary Data 7 S4). The L0Reg framework showed similar performance to GENIE3 on DREAM4 (Supplementary Figure 5a), but significantly outperformed GENIE3 on GNW-100 datasets (Supplementary Figure 5b).

Supplementary Tables

Supplementary Table 1: The IR genes were initially screened according to the differential expression analysis. The differential expression analysis by DESeq2 was performed on 249 IR genes, and finally the 42 eligible genes were derived. The values are the Log2FoldChange of the gene in TCGA-LUAD clusters compared with GTEx.

Gene	Cluster 1	Cluster 2	Cluster 3	Cluster 4	Label
ARG2	1.068748498	1.435370977	0.781048211	2.336935524	Cluster 1,2,4 Up
B2M	-0.004211191	-0.104926564	0.114397713	0.392795512	Cluster 1,2,3,4 Down
BTLA	0.990669059	0.800643626	1.364668557	0.982979059	Cluster 3 Up
CCL17	1.383106019	0.801210085	2.013125693	-0.032420549	Cluster 2,4 Down
CCL20	1.033162703	0.329359631	0.430207709	2.166989898	Cluster 2,3 Down
CCL22	2.783985663	2.117572566	2.572143819	3.027702314	Cluster 1,2,3,4 Up
CCL26	0.252332568	0.174471436	-0.597943389	1.020385309	Cluster 1,2,3 Down
CCL28	2.289901968	1.663388158	1.889566644	1.444766738	Cluster 1,2,3,4 Up
CCL7	1.455509351	1.05992542	0.713530321	0.742279736	Cluster 3,4 Down
CCR5	1.084308482	0.840983441	1.084063482	0.897106605	Cluster 2,4 Down
CCR6	2.387655398	1.715356466	2.806253093	2.154082808	Cluster 1,2,3,4 Up
CCR7	1.173137286	0.777141464	1.641206615	0.981187532	Cluster 2,4 Down
CD1C	1.680133462	0.938060174	2.25464767	0.905251004	Cluster 2,4 Down
CD2	1.069928246	0.878039515	1.269804249	1.103914888	Cluster 2 Down
CD3D	1.19336619	0.972103177	1.374274193	1.385861692	Cluster 2 Down
CD40	-0.060398417	-0.286279216	-0.0341285	-0.559433302	Cluster 1,2,3,4 Down
CD70	1.48932161	1.556479622	1.190222091	0.953923521	Cluster 4 Down
CXCL17	1.987389828	2.229149265	2.049345273	2.588896741	Cluster 1,2,3,4 Up
CXCL5	0.041471029	-0.380748504	-0.863972171	1.537799189	Cluster 1,2,3 Down
CXCL6	1.093426514	-0.318544613	1.542844127	0.263673742	Cluster 2,4 Down
CXCR5	0.807416586	0.817415989	1.368799407	0.476618845	Cluster 1,2,4 Down
EZH2	1.582946797	1.689074225	0.837558807	1.874686769	Cluster 1,2,4 Up
HMGB1	0.523369858	0.747919243	0.333826936	1.048596132	Cluster 1,2,3 Down
HSP90AA1	0.58209368	0.855811417	0.351739817	1.150495005	Cluster 1,2,3 Down
HSP90AB1	1.054429207	1.098557141	0.679888016	1.432300582	Cluster 3 Down
HSP90B1	0.881135357	1.136724252	0.641758066	1.993779049	Cluster 1,3 Down
HSPA13	1.165742613	1.326465692	0.800474889	1.639924689	Cluster 3 Down
HSPA14	0.756833763	0.964939145	0.479282955	1.409786599	Cluster 1,2,3 Down
HSPA4	1.064874491	1.177033749	0.809804931	1.49142851	Cluster 3 Down
HSPA5	1.079164266	1.210366475	0.873827142	1.75320347	Cluster 3 Down
HSPA8	0.993251117	0.979022802	0.718326749	1.379033734	Cluster 1,2,3 Down

HSPA9	0.713429445	0.883314593	0.426479516	1.393511922	Cluster 1,2,3 Down
IFNB1	1.600301407	0.961143725	0.864340879	0.427461334	Cluster 2,3,4 Down
IL4	0.36267112	0.647484535	0.602384807	1.223424361	Cluster 4 Up
KLRK1	-1.943328823	-1.812264452	-1.746682315	-1.694690979	Cluster 1,2,3,4 Down
LAG3	0.70067676	0.95701689	0.581617999	1.108816524	Cluster 4 Up
MDM2	0.383520829	0.416533627	0.590131194	1.005402538	Cluster 1,2,3 Down
NT5E	3.375714618	2.69881522	3.10853904	2.732597974	Cluster 1,2,3,4 Up
RAET1G	0.435759321	0.660525256	0.421711803	2.05258578	Cluster 1,2,3 Down
SMC3	0.934301028	1.078798537	0.474207121	1.319442271	Cluster 2,4 Up
TAP1	0.640150316	0.54062252	0.441464309	0.879042226	Cluster 1,2,3,4 Down
TLR10	0.843568361	0.477163835	1.610190189	1.161635449	Cluster 1,2 Down

Supplementary Table 2: 5 datasets provided in DREAM4 and 10 datasets generated by GNW-100.

Source	No. of datasets	No. of samples	No. of genes	Type
DREAM4	5	100	100	Simulation
GNW-100	10	100	100	Simulation

Supplementary Figures

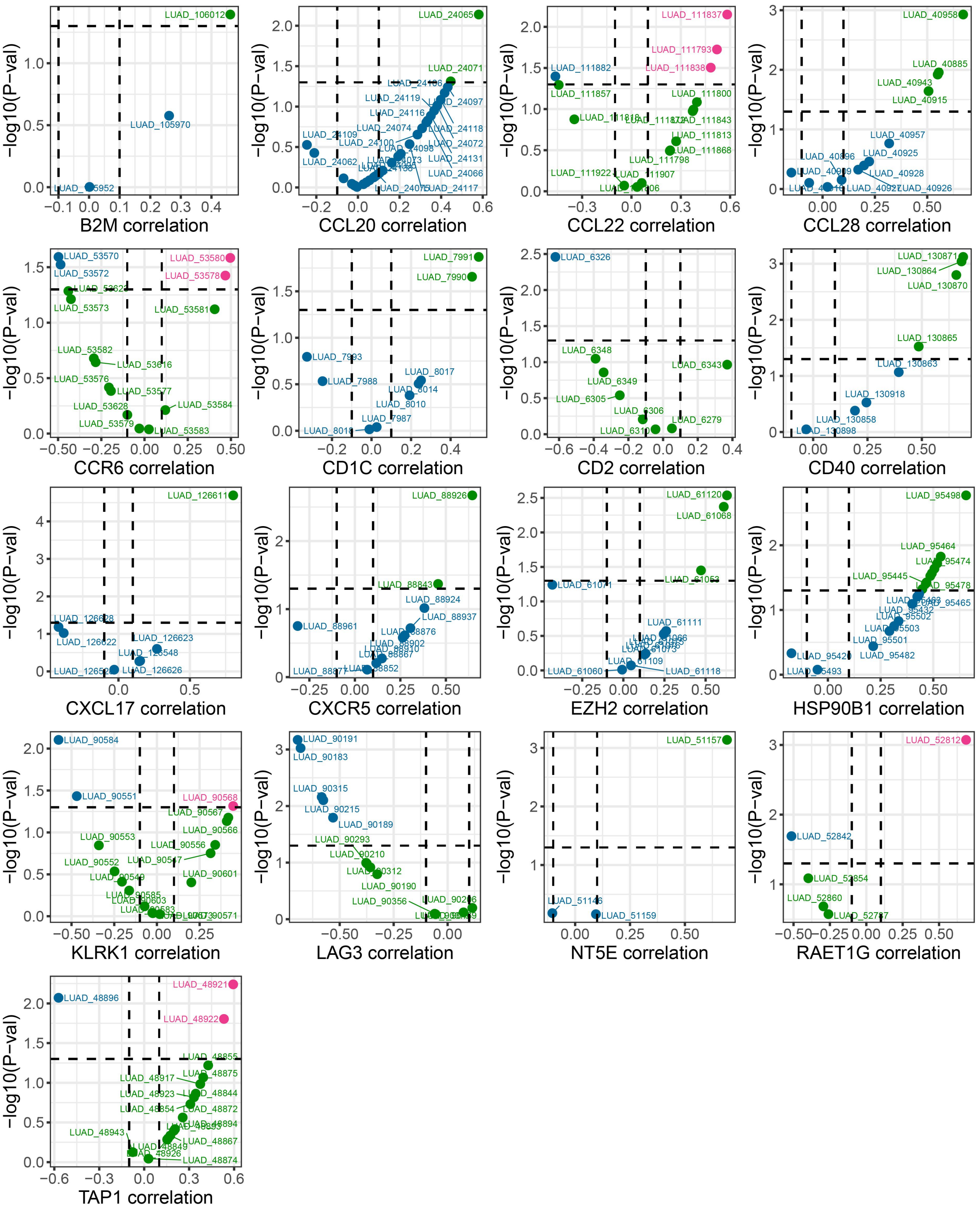

Supplementary Figure 1: The peak signals correlation with 17 IR genes.

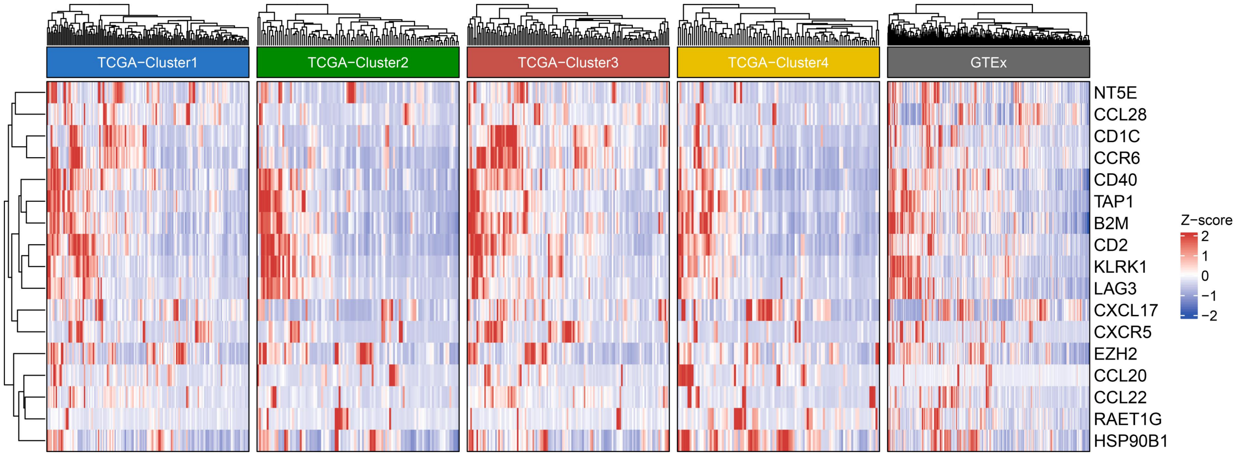

Supplementary Figure 2: The expression heatmap of 17 IR genes in 4 clusters of TCGA-LUAD and GTEx samples. Each row denotes the IR gene and each column represents a sample. Note that the expression levels are converted to Z-score (-2 to 2).

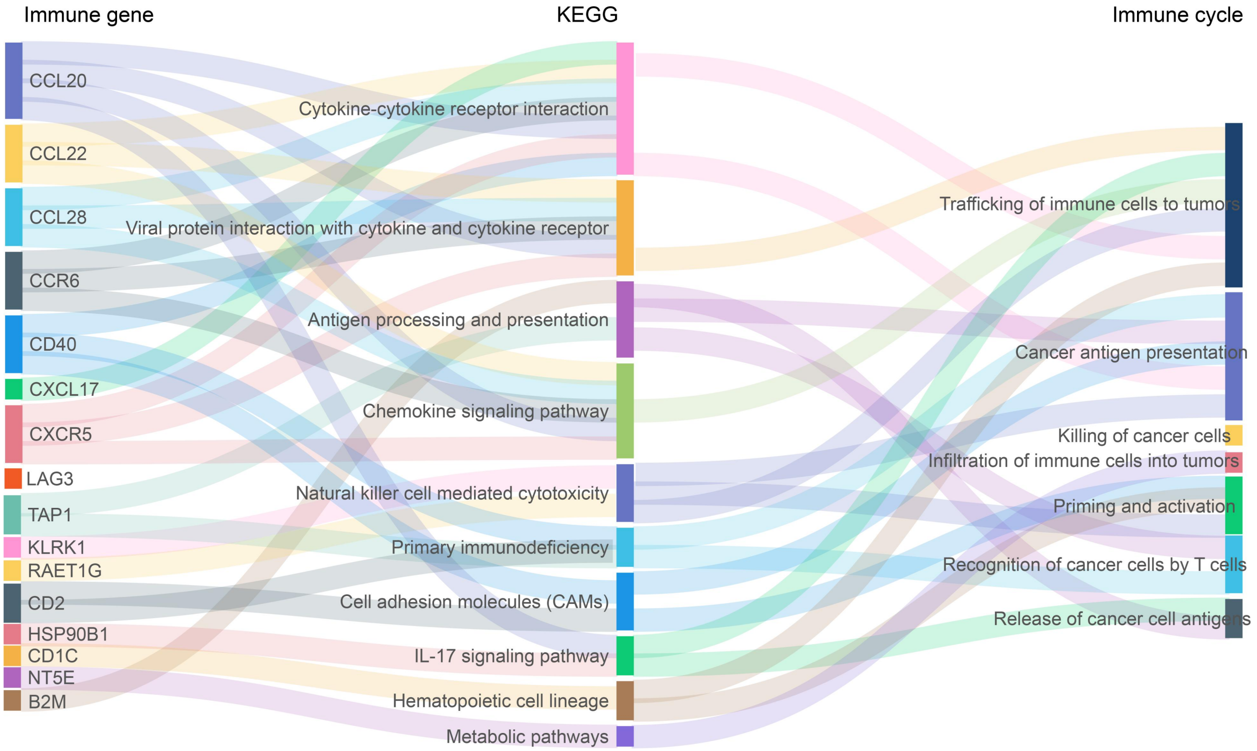

Supplementary Figure 3: The relationship between KEGG enrichment analysis results of IR genes and immune cycle.

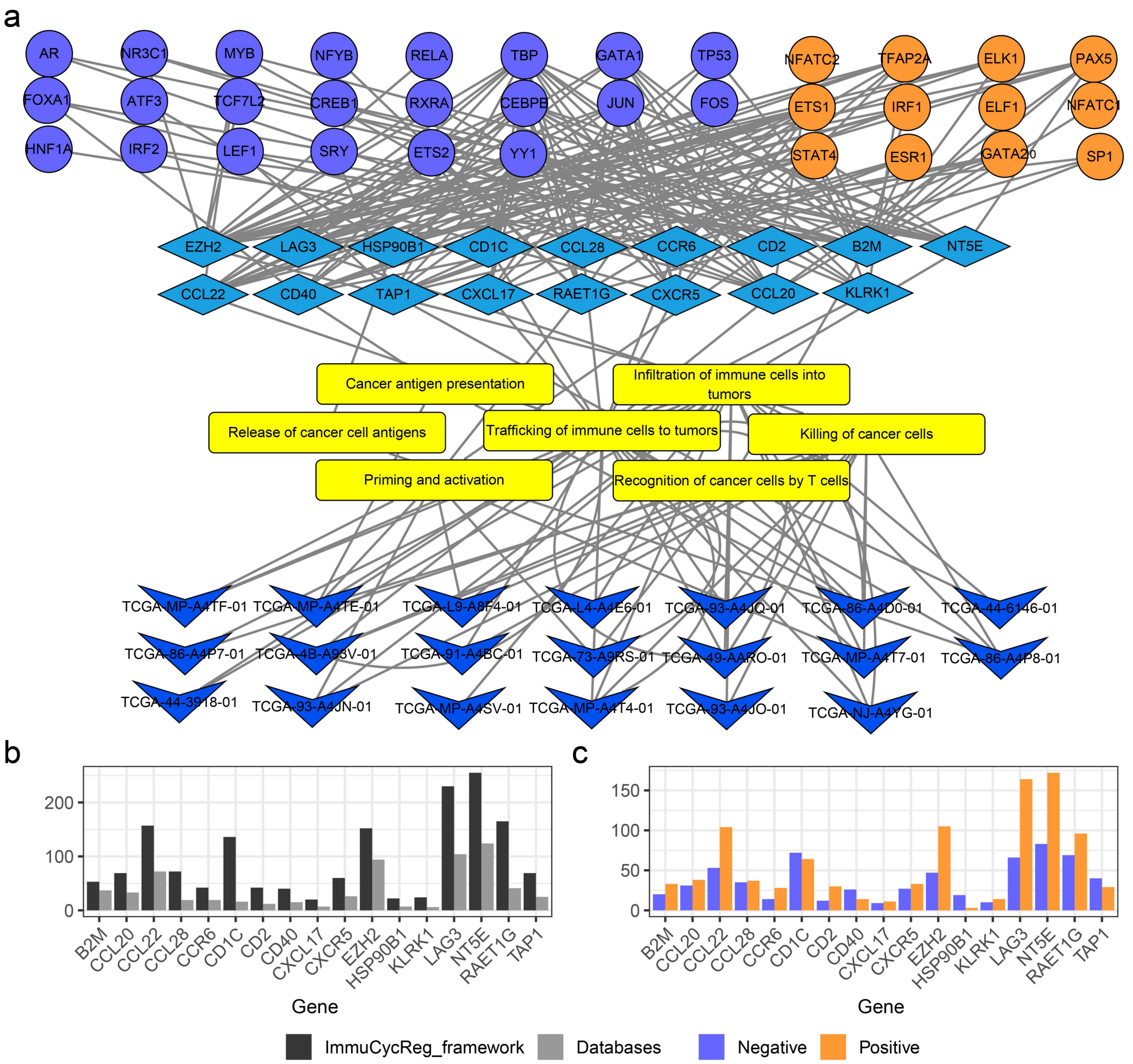

Supplementary Figure 4: (**a**) The regulatory network inferred by the ImmuCycReg framework on single sample level. (**b**) The number of regulatory relationships speculated by the ImmuCycReg framework compared with databases (The JASPAR[10], ENCODE[11], ChEA[12], MotifMap[13], and TRANSFAC[14]). (c) The number of positive and negative regulatory edges of the regulatory network for each IR gene.

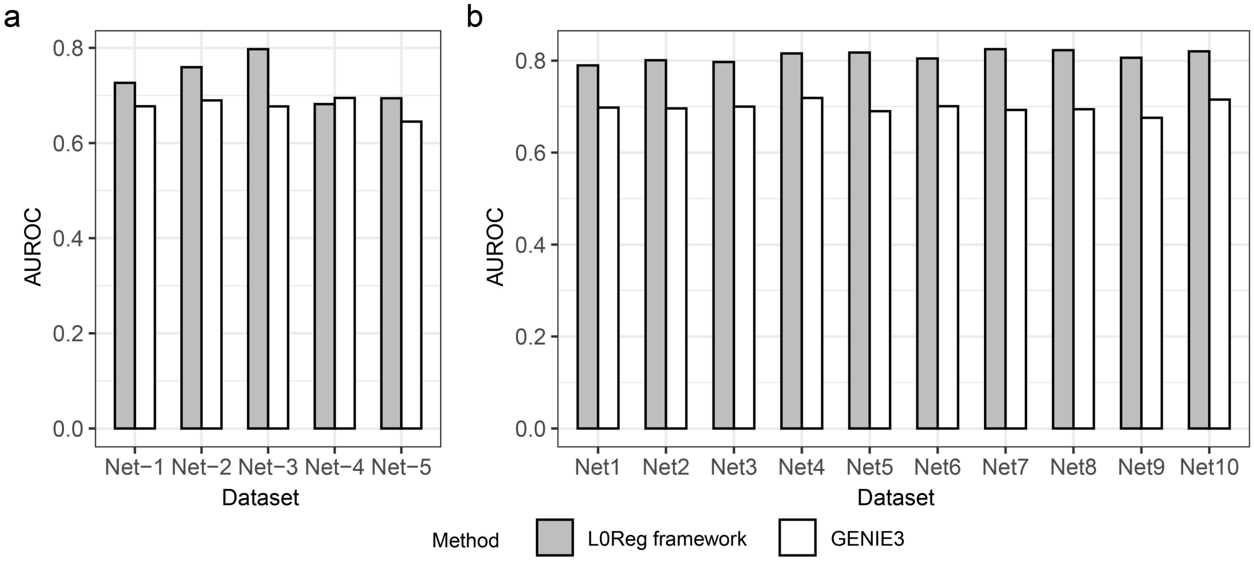

Supplementary Figure 5: (**a**) The AUROC values of the L0Reg framework and GENIE3 on DREAM4 datasets (from Net-1 to Net-5). (**b**) The AUROC values of the L0Reg framework and GENIE3 on GNW-100 datasets (from Net1 to Net10). The higher the AUROC value, the better the performance.

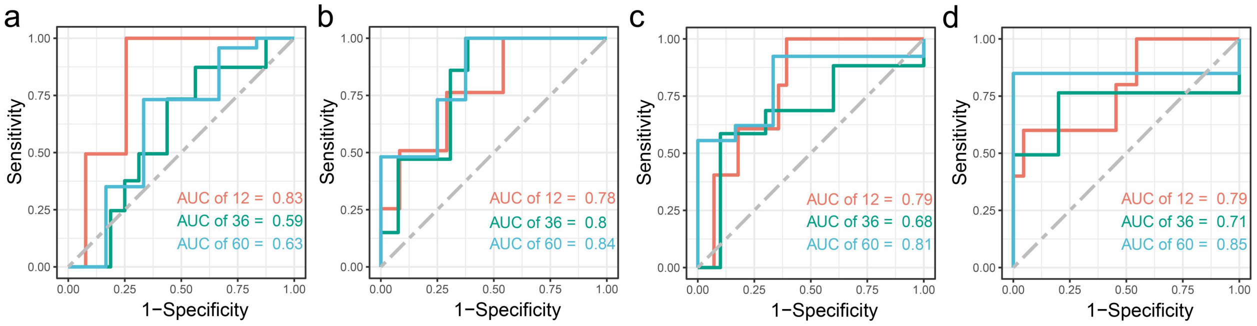

Supplementary Figure 6: (**a**) (**b**) (**c**) and (**d**) The area under curve (AUC) of the Lasso-Cox models of Cluster 1, 2, 3 and 4 on the test datasets, respectively. Each model predicted patient 1-, 3-, and 5-year (the time unit in the figure is month) Overall Survival (OS) based on the risk factors in Supplementary Figure 7a.

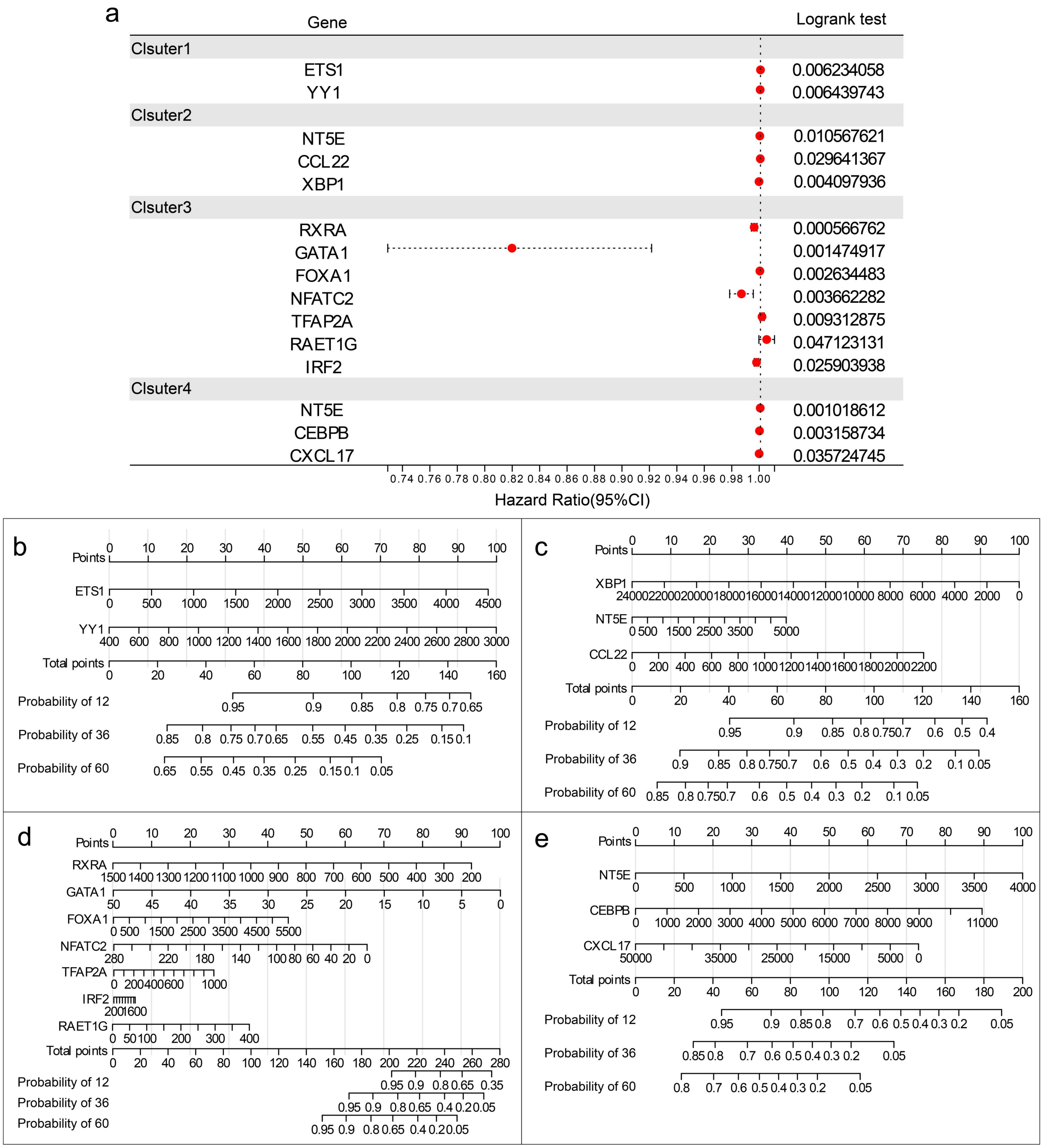

Supplementary Figure 7: (**a**) The *P*-value of IR genes and TFs used in Lasso-Cox. (**b**) (**c**) (**d**) and (**e**) The nomogram of 4 TCGA-LUAD subtypes based on the risk factors.

Supplementary Data

Supplementary Data 1: 2230 IR genes for NMF clustering.

Supplementary Data 2: 249 immune cycle signature genes.

Supplementary Data 3: Results of NMF clustering, TCGA-LUAD samples and GTEx samples.

Supplementary Data 4: Results of DESeq2 differential expression analysis between TCGA-LUAD subtypes and GTEx samples.

Supplementary Data 5: Results of ClueGO enrichment analysis

Supplementary Data 6: Sequences, peaks and TFs of IR genes exported from the ImmuCycReg framework

Supplementary Data 7: Results of cross validation for the L0Reg framework

Supplementary Data 8: Results of the regulatory network derived from the ImmuCycReg framework and L0Reg framework.

Supplementary Data 9: Results of Risk Score.

Supplementary References

1. Gaujoux, R. & Seoighe, C. A flexible R package for nonnegative matrix factorization. *BMC Bioinformatics* **11**, 367 (2010).
2. Love, M. I., Huber, W. & Anders, S. Moderated estimation of fold change and dispersion for RNA-seq data with DESeq2. *Genome Biol.* **15**, 550 (2014).
3. Pavlidis, P. & Noble, W. S. Analysis of strain and regional variation in gene expression in mouse brain. *Genome Biol.* **2**, 1–15 (2001).
4. Huynh-Thu, V. A., Irrthum, A., Wehenkel, L. & Geurts, P. Inferring Regulatory Networks from Expression Data Using Tree-Based Methods. *PLoS ONE* **5**, e12776 (2010).
5. Potier, D. *et al.* Mapping Gene Regulatory Networks in Drosophila Eye Development by Large-Scale Transcriptome Perturbations and Motif Inference. *Cell Rep.* **9**, 2290–2303 (2014).
6. Chandrasekaran, S. *et al.* Behavior-specific changes in transcriptional modules lead to distinct and predictable neurogenomic states. *Proc. Natl. Acad. Sci.* **108**, 18020–18025 (2011).
7. Marbach, D. *et al.* Wisdom of crowds for robust gene network inference. *Nat. Methods* **9**, 796–804 (2012).
8. Marbach, D., Schaffter, T., Mattiussi, C. & Floreano, D. Generating Realistic *In Silico* Gene Networks for Performance Assessment of Reverse Engineering Methods. *J. Comput. Biol.* **16**, 229–239 (2009).
9. Schaffter, T., Marbach, D. & Floreano, D. GeneNetWeaver: in silico benchmark generation and performance profiling of network inference methods. *Bioinformatics* **27**, 2263–2270 (2011).
10. Portales-Casamar, E. *et al.* JASPAR 2010: the greatly expanded open-access database of transcription factor binding profiles. *Nucleic Acids Res.* **38**, D105-110 (2010).
11. ENCODE Project Consortium. A user's guide to the encyclopedia of DNA elements (ENCODE). *PLoS Biol.* **9**, e1001046 (2011).
12. Lachmann, A. *et al.* ChEA: transcription factor regulation inferred from integrating genome-wide ChIP-X experiments. *Bioinforma. Oxf. Engl.* **26**, 2438–2444 (2010).

13. Daily, K., Patel, V. R., Rigor, P., Xie, X. & Baldi, P. MotifMap: integrative genome-wide maps of regulatory motif sites for model species. *BMC Bioinformatics* **12**, 495 (2011).

14. Matys, V. *et al.* TRANSFAC: transcriptional regulation, from patterns to profiles. *Nucleic Acids Res.* **31**, 374–378 (2003).
